# Hydrogen Sulfide Promotes Adipogenesis in 3T3L1 Cells

**DOI:** 10.1371/journal.pone.0119511

**Published:** 2015-03-30

**Authors:** Chin-Yi Tsai, Meng Teng Peh, Wei Feng, Brian William Dymock, Philip Keith Moore

**Affiliations:** 1 Neurobiology Group, Life Science Institute, Department of Pharmacology, Yong Loo Lin School of Medicine, Singapore, Singapore; 2 Department of Pharmacy, Faculty of Science, National University of Singapore, Singapore, Singapore; University of Calgary, CANADA

## Abstract

The effect of hydrogen sulfide (H_2_S) on differentiation of 3T3L1-derived adipocytes was examined. Endogenous H_2_S was increased after 3T3L1 differentiation. The expression of the H_2_S-synthesising enzymes, cystathionine γ-lyase (CSE), cystathionine β-synthase (CBS) and 3-mercaptopyruvate sulfurtransferase (3-MST), was increased in a time-dependent manner during 3T3L1 differentiation. Expression of genes associated with adipogenesis related genes including fatty acid binding protein 4 (FABP4/aP2), a key regulator of this process, was increased by GYY4137 (a slow-releasing H_2_S donor compound) and sodium hydrosulfide (NaHS, a classical H_2_S donor) but not by ZYJ1122 or time-expired NaHS. Furthermore expression of these genes were reduced by aminooxyacetic acid (AOAA, CBS inhibitor), DL-propargylglycine (PAG, CSE inhibitor) as well as by CSE small interference RNA (siCSE) and siCBS. The size and number of lipid droplets in mature adipocytes was significantly increased by both GYY4137 and NaHS, which also impaired the ability of CL316,243 (β3-agonist) to promote lipolysis in these cells. In contrast, AOAA and PAG had the opposite effect. Taken together, we show that the H_2_S-synthesising enzymes CBS, CSE and 3-MST are endogenously expressed during adipogenesis and that both endogenous and exogenous H_2_S modulate adipogenesis and adipocyte maturation.

## Introduction

Obesity, a major health issue in developed countries, is now widely regarded as a chronic inflammatory state which contributes to numerous pathologies including dyslipidemia, coronary heart disease, non-alcoholic fatty liver, insulin resistance and type II diabetes [1–4]. Obesity is associated with accumulation of excess triacylglyceride (TG) in adipocytes either due to innate hyperadipogenesis or to lipid overloading in adipose tissue [5–7]. Lipid accumulation in adipose tissue is tightly controlled by a range of adipogenesis-related molecules including fatty acids binding protein 4 (FABP4/aP2), peroxisome proliferator-activated receptor γ (PPARγ), CCAAT/enhancer binding protein α (CEBPα), sterol regulatory element binding protein-1 (SREBP1), carbohydrate responsive element binding protein (ChREBP), fatty acid synthase (FAS), hormone-sensitive lipase (HSL), perilipin A and a 47 kDa tail interacting protein (TIP47) [8–12]. Among these, PPARγ and CEBPα are particularly important in the early stages of adipogenesis since they stimulate FABP4/aP2 thereby activating FABP4/aP2 to trigger downstream FAS, ChREPB and SREBP1 mRNA activation and thus promote adipocyte maturation [8,13,14]. Moreover, HSL, perilipin A and TIP47, enzymes which bind intracellular lipid droplets, serve to regulate TG breakdown and glycerol release from mature adipocytes [13,15].

Hydrogen sulfide (H_2_S) is generated from L-cysteine by cystathionine γ lyase (CSE) or cystathionine β-synthase (CBS) and from 3-mercaptopyruvate by 3-mercaptopyruvate sulfurtransferase (3-MST) [16,17]. These enzymes occur widely in mammalian cells and tissues and produce H_2_S which, in turn, plays multiple roles in regulating cardiovascular function, inflammation, insulin resistance and glucose metabolism [18–23]. It has recently been reported that H_2_S is also formed in fat tissues [21,24] and that H_2_S can impair insulin signaling and glucose uptake into adipocytes [21–24]. Moreover, H_2_S reportedly reduces insulin resistance in adipocytes from obese mice fed a high fat diet [24]. Together, these studies suggest a role for adipose H_2_S in insulin resistance and glucose homeostasis [24]. However, the precise biological effect of either endogenous or exogenous H_2_S on adipocytes and the contribution which this gas makes on adipogenesis are not clear. With this in mind, we have now used both fast- (NaHS) and slow-releasing (GYY4137) H_2_S donors, and for comparison, time-expired NaHS and ZYJ1122 (a structural analogue of GYY4137 lacking sulfur and thence unable to release H_2_S), drugs which inhibit endogenous H_2_S biosynthesis (AOAA, PAG) as well as siCBS and siCSE as tools to assess the effect of H_2_S on adipocyte biology *in vitro*.

## Materials and Methods

### Reagents, drugs and antibodies

Dulbecco’s modified Eagle’s medium (DMEM) and fetal bovine serum (FBS) were purchased from Gibco^@^ (Grand Island, NY). Growth medium comprising DMEM containing 15% w/v FBS and penicillin (100 U/ml)/streptomycin (100 μg/ml) and differentiation medium consisting of DMEM containing 15% w/v FBS, penicillin (100 U/ml)/streptomycin (100 μg/ml), 1-methyl-3-isobutylxanthine (MIX, 0.5 mM), dexamethasone (DEX, 0.5 μM) and insulin (1.7 μM) were prepared. Phosphate buffered saline (PBS) containing 1% w/v Triton X-100 (1% v/v), sodium chloride (NaCl, 250 mM), Tris hydrochloride (50 mM, pH7.5), ethylenediaminetetraacetic acid (EDTA, 5 mM), leupeptin (1 μg/ml), aprotinin (10 μg/ml) and phenylmethylsulfonyl fluoride (PMSF, 1 mM) was used to lyse cells. Lipid droplets in cells were stained using Oil red O (Cayman chemicals, USA). Adipocyte lipolysis was stimulated with CL-316243 (β3-adrenoceptor agonist, 5 nM). NaHS, AOAA and PAG were purchased from Sigma Aldrich (St. Louis, MO). Time-expired NaHS was prepared by exposing a solution of NaHS (50 μM) to the air for 18 h. ZYJ1122 and GYY4137 were provided by Professor Tan Choon-Hong (Department of Chemistry, Nanyang Technological University, Singapore). For siRNA knockdown experiments, CSE siRNA (siCSE), CBS siRNA (siCBS) and Lipofectamine^3000^ were purchased from Life Technologies (Paisley, UK). For western blotting, goat anti-mouse FABP4/aP2 antibody was purchased from Santa Cruz Biotechnology (Santa Cruz, CA), rabbit anti-mouse CBS, β-actin and mouse anti-mouse CSE were obtained from Abcam (Cambridge, MA), and rabbit anti-mouse 3-MST was obtained from Sigma-Aldrich (St. Louis, MO). Downstream metabolites of lipolysis and the released glycerol were detected by colorimetry using a lipase-based adipolysis assay kit was purchased from Cayman chemicals (Ann Arbor, MI, USA).

### Cell Culture

3T3L1, a fibroblast-like mouse preadipocyte cell line, was purchased from the American Type Culture Collection (ATCC). Frozen 3T3L1 cells were recovered and incubated (37°C, 5% CO_2_) in DMEM containing 10% w/v FBS until confluency. Confluent 3T3L1 cells were then incubated in differentiation medium for 3 days after which medium was aspirated and growth medium added for an additional 4 days to allow 3T3L1 cells to differentiate into mature adipocytes. The medium was changed every 2 days until cells were fully differentiated.

### Measurement of H_2_S concentration

The H_2_S concentration in cultured medium was assessed using a sulfonyl azide-based fluorescent probe, 2,6-dansyl azide, as described elsewhere [25]. Briefly, confluent 3T3L1 cells were cultured in differentiating medium and incubated with or without GYY4137 (50 μM) or NaHS (50 μM). After incubation (48 h), cells were collected and medium mixed (50% v/v) with 2,6-dansyl azide probe solution (0.5 ml of 0.4 mM in 90% v/v MeCN in pH 7.4 PBS mixture (‘buffer solution’)) in 4 ml screw-cap glass vials. Fluorescence was read in a SpectraMax M3 Microplate reader at EX325\EM450 in triplicate. The samples were kept at 37°C in the dark. Readings were compared with a standard curve generated from a 400 μM stock solution of probe in buffer solution, dispensed into 15 x 4 ml screw-cap glass vials (1.5 ml stock solution per vial). A 400 μM solution of sodium sulfide (Na_2_S) in buffer solution was added to the probe solution and the total volume made up to 3 ml with buffer solution so that the final probe concentration was 200 μM, and the final Na_2_S concentrations were 0, 50, 100, 150 and 200 μM. Fluorescence readings (gain = 80) were recorded for 3 samples from each vial.

### Western Blotting

Adipocytes were rinsed once with ice-cold PBS, lysed with lysis buffer on ice (10 min) and the lysate centrifuged (12000*g*, 5 min, 4°C). Protein concentration was then determined by the Bradford reaction (Bio-Rad Ltd., California, USA). Aliquots (50 μg) of cell suspension were resolved in 8% or 12% SDS-polyacrylamide gels and transferred onto nitrocellulose membranes (Bio-Rad Ltd., California, USA). After blocking (1 h) with 5% w/v skimmed milk in 0.1% w/v Tween/PBS, blots were incubated with the appropriate primary antibodies and then with HRP-conjugated secondary antibodies. Blots were detected using enhanced chemiluminescent reagent (Merck Millipore Ltd., USA) and quantified using Image J software (National Institutes of Health, Bethesda, MD, USA).

### mRNA extraction and quantitative real-time PCR

Genomic mRNA was collected from 3T3L1 cells which had been incubated with or without GYY4137, NaHS, AOAA or PAG for 48 h. Cultured cells were washed with ice cold PBS and incubated with TRIzol^@^ reagent (Invitrogen, Carlsbad, CA) for 1 min. RNA was extracted using Aurum Total RNA Mini Kits (Bio-Rad, Hercules, CA) and 1 μg of total RNA was transcribed into cDNA using an iScript cDNA Synthesis kit (Bio-Rad Laboratories, Hercules, CA), according to the manufacturer’s protocols. Relative quantitative real-time PCR was performed by administering 3 μl of cDNA, 2 μl of primers to 5 μl of the reaction mix buffer from the Power SYBR Green PCR master mix kit (Life Technologies, Paisley, UK), the amplification reaction was monitored using a ViiA7 qPCR thermal cycler (Applied Biosystem, Paisley, UK). Expression values were determined by 2^-ΔΔCT^ equation and normalized with 18S housekeeping gene. The specific primers for representative genes are listed in Table 1.

**Table 1:** Primer sequences of adipogenesis-related genes.

Category	Genes name		Sequence	References
**Adipocyte differentiation factors**	FABP4/aP2	-F	5′-TGGAAGCTTGTCTCCAGTGA-3′	[26]
		-R	5′-AATCCCCATTTACGCTGATG-3′	
	PPARγ	-F	5′-CAAGAATACCAAAGTGCGATCAA-3′	[26]
		-R	5′-GAGCAGGGTCTTTTCAGAATAATAAG-3′	
	CEBPα	-F	5′-TGGACAAGAACAGCAACGAGTA-3′	[27]
		-R	5′-GCAGTTGCCATGGCCTTGA-3′	
**Adipognesis transcription factors**	FAS	-F	5′-GGTCGT TTCTCCATTAAATTCTCA T-3′	[28]
		-R	5′-CCT TCTAAAGACCCTTTCAAAGAT C-3′	
	ChREBP	-F	5′-GTCCGATATCTCCGACACACTCTT-3′	[29]
		-R	5′-CATTGCCAACATAAGCATCTTCTG-3′	
	SREBP1	-F	5′-GGAGCCA-TGGATTGCACATT-3′	[30]
		-R	5′-AGGCCAGGGAAGT-CACTGTCT-3′	
**Lipolysis related enzymes**	HSL	-F	5′-GCTGGGCTGTCAAGCACTGT-3′	[28]
		-R	5′-TACCGTCGGATGGGTCAATG-3′	
	Perilipin A	-F	5′-GGCCTGGACGACAAAACC-3′	[31]
		-R	5′-CAGGATGGGCTCCATGAC-3′	
	TIP47	-F	5′-GGAACTGGTGTCATCAACAG-3′	[32]
		-R	5′-GGT CAC ATC CAC TGC TCC TG-3′	
**Internal control**	18S	-F	5′-TAAGTCCCTGCCCTTGGTACACA-3′	[33]
		-R	5′-GATCCGAGGGCCTCACTAAAC-3′	

-F for forward primer sequence

-R for reverse primer sequence.

### Transfection of adipocytes by small interference RNA (si-RNA)

3T3L1 cells were cultured in differentiation medium and transfected with either siCSE or siCBS siRNAs using Lipofectamine^TM^ 3000 for 72 h. Briefly, and according to the manufacturer’s instructions, 250 μl of Lipofectamine^TM^ 3000 only or siCSE (5 μg) or siCBS (5 μg):Lipofectamine 3000 complexes were mixed at room temperature for 5 min and then added to 50% confluent 3T3L1 cells for 72 h prior to Western Blotting of CSE, CBS and FABP4/aP2 as described above.

### Measurement of cell lipolysis and release of glycerol in mature 3T3L1 derived adipocytes

Mature adipocytes were pre-treated with GYY4137 (50 μM), NaHS (50 μM), AOAA (1 mM), PAG (10 mM) for 2 h. After washing with PBS, cells were treated with 5 nM CL316,243 (to stimulate adipocytes releasing glycerol) and co-incubated with or without GYY4137, NaHS, AOAA or PAG in Krebs Ringer Buffer (KRB) (13 mM NaCl, 4.7 mM KCl, 2.5 mM MgSO_4_, 3.3 mM CaCl_2_, 24.5 mM NaHCO_3_, 1 mM KH_2_PO_4_, 5 mM glucose, 3% w/v bovine serum albumin) for 1 h. Buffer was then collected and the release of glycerol determined using an Adipolysis assay kit (Cayman Chemicals, MI, USA).

### Oil-Red O Staining

Mature adipocytes were fixed in 24 well plates with 4% w/v paraformaldehyde (Sigma-Aldrich, St. Louis, MO) for 30 min at room temperature. Fixed cells were washed with PBS and then stained (15 min) with Oil Red O (stock solution: 3 mg/ml dissolved in isopropanol; working solution: 60% Oil Red O stock solution and 40% distilled water) and the counterstain, hemotoxylin (Sigma-Aldrich, St. Louis, MO). Lipid density was analyzed at 540 nm using a Bio-Tek plate reader (BioTek. Instruments Inc., Winooski, VT, USA).

### Statistical analysis

All experiments were performed on at least four separate occasions and quantitative data is expressed as mean±SEM. Statistical significance was determined by One-Way ANOVA followed by Fisher's least significant difference (LSD) posthoc analysis. SPSS version 21 (SPSS Inc., Chicago, IL) was used for analysis. Statistical significance was set at *P* <0.05.

## Results

### Expression of CBS, CSE and 3-MST during adipocyte differentiation

**Fig 1:**
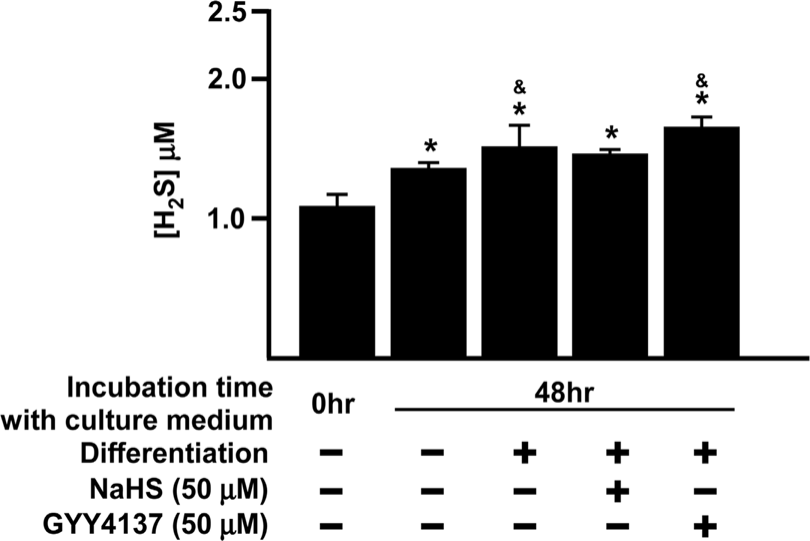
Increase in H_2_S concentration in culture medium after differentiation. Confluent 3T3L1 cells were washed with PBS (37°C) and resuspended in normal growth DMEM for 10 min. Aliquots of medium were collected (‘baseline’ group). 3T3L1 cells were then cultured for 2 days either in DMEM (‘negative control’ group) or differentiation medium (‘positive’ control group). To test the effect of H_2_S releasing drugs, 3T3L1 cells were co-incubated with GYY4137 (50 μM) or NaHS (50 μM) for 2 days. H_2_S concentration in the medium was detected using a 2,6-dansyl azide fluorescent probe. Statistical significance was determined by ANOVA followed by Fisher's LSD posthoc analysis. Data shown are mean±SEM of 4 independent experiments. *P < 0.05 vs. baseline group; &P < 0.05 vs. 48 h negative control group.

**Fig 2:**
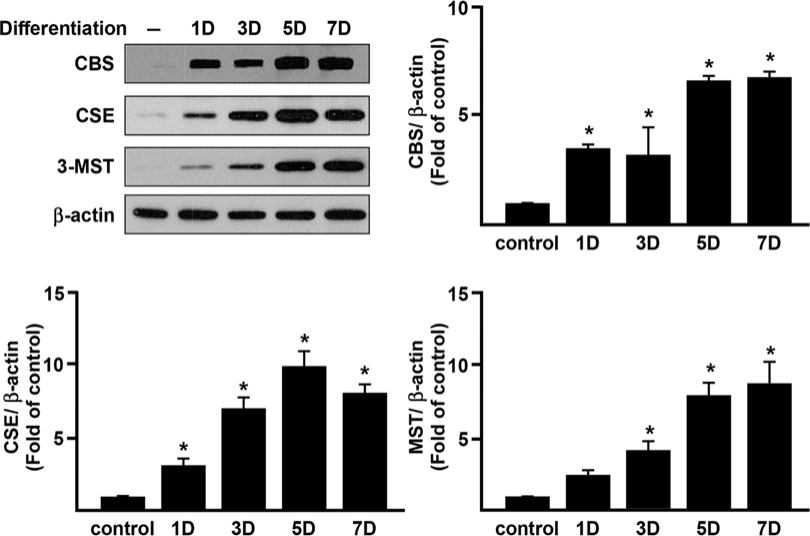
Upregulation of CBS, CSE and 3-MST during differentiation of 3T3L1 cells. H_2_S-synthesising enzyme (CBS, CSE and 3-MST) expression during adipocyte differentiation was determined by Western Blotting. Data shows protein expression compared to the negative control group (normalized to β-actin) set as 1. Statistical significance was determined by ANOVA followed by Fisher's LSD posthoc analysis. Data shown are mean±SEM from 4 independent experiments. *P < 0.05 vs. negative control group.

We first determined whether H_2_S was generated naturally during adipocyte differentiation (Fig 1) and whether the expression of CBS, CSE and 3-MST was altered during the process (Fig 2).

H_2_S concentration in the culture medium was significantly increased after 48 h of differentiation whilst inclusion of GYY4137, but not NaHS, into the medium caused a small, but statistically significant, increase in H_2_S concentration (Fig 1). CBS expression increased after differentiation for 1 day and plateaued at day 5–7 just prior to cells reaching full maturity (Fig 2). CSE and 3-MST expression increased steadily from day 1 to day 7 (Fig 2). These data suggest that H_2_S, generated by the activity of either CBS, CSE or 3-MST or a combination thereof, may have functional role(s) to play in adipocytes.

### H_2_S upregulates adipogenesis-related genes

**Fig 3:**
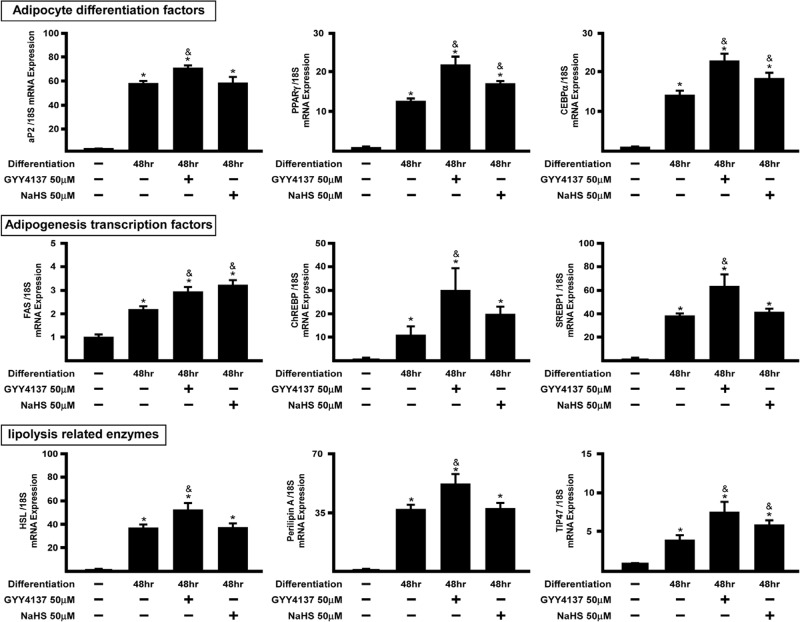
H_2_S upregulates adipogenesis-related genes. 3T3L1 cells were co-incubated with GYY4137 (50 μM) or NaHS (50 μM) for 2 days and subjected to real-time PCR assay of genes relevant to lipid metabolism. Statistical significance was determined by ANOVA followed by Fisher's LSD posthoc analysis. Data shown are mean±SEM of 6 independent experiments. *P < 0.05 vs. negative control group; ^&^P < 0.05 vs. 48 h positive control group.

The maturation of adipocytes is tightly regulated by adipocyte differentiation factors, adipogenesis transcription factors and lipolysis related enzymes [7–9,14]. To determine the effect of H_2_S on adipogenesis, confluent adipocytes in normal growth medium (i.e. negative control group), cells with differentiation medium (i.e positive control group) and cells co-incubated with differentiation medium containing either GYY4137 (50 μM) or NaHS (50 μM) were incubated for 48 h. PPARγ and CEBPα, mRNA expression was induced by both GYY4137 and NaHS (Fig 3).

**Fig 4:**
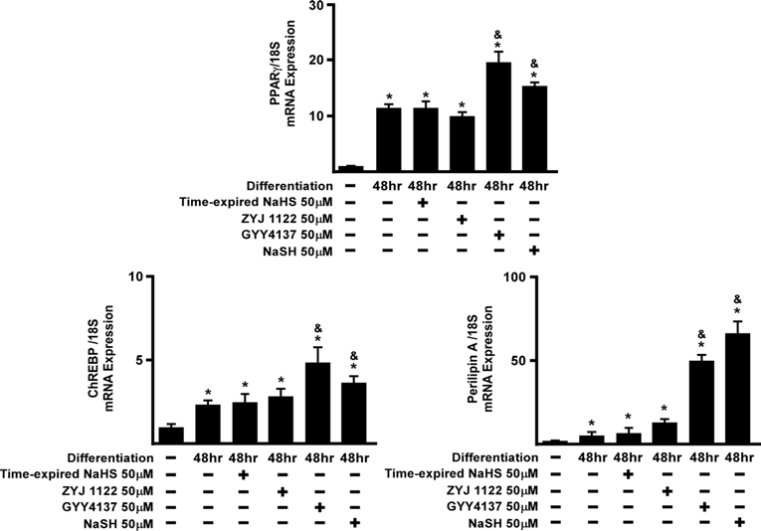
Effect of H_2_S releasing drugs on expression of markers of adipogenesis. 3T3L1 cells were co-incubated with GYY4137 (50 μM), NaHS (50 μM), time-expired NaHS (50 μM note: which was left at room temperature for 18 h and without H_2_S-releasing activity) or ZYJ1122 (50 μM), for 2 days and subject to real-time PCR assay of genes relevant to adipogenesis. Statistical significance was determined by ANOVA followed by Fisher's LSD posthoc analysis. Data shown are mean±SEM of 4 independent experiments. *P < 0.05 vs. negative control (non-differentiated) group; ^&^P < 0.05 vs. 48 h positive control group.

GYY4137 (but not NaHS) also induced expression of FABP4/aP2, ChREBP, SREBP1, HSL and perilipin A mRNA expression after 48 h incubation. Among the H_2_S responsive genes, adipogenesis transcription factors (FAS, ChREBP and SREBP1c), are responsible for promoting adipocyte formation [12,14,34], whilst the lipolysis related enzyme genes (HSL, TIP47 and perilipin A), play significant roles in facilitating TG breakdown into glycerol and free fatty acids (FAA) in mature adipocytes [6,7,35,36]. Expression of FAS and TIP47 mRNA was significantly enhanced in both GYY4137- and NaHS-treated groups (c.f. positive control group, P< 0.05). To determine whether these effects of GYY4137 were indeed due to H_2_S release we conducted control experiments in which 3T3L1 cells were treated with either time-expired NaHS (50 μM) or ZYJ1122 (50 μM; a GYY4137 analogue lacking sulfur and hence incapable of releasing H_2_S (for chemical structure, see [37]) during adipocyte differentiation (Fig 4).

Expression of PPARγ, ChREBP and Perilipin A mRNA was increased by GYY4137 and NaHS (Fig 4) but not time-expired NaHS or ZYJ1122. GYY4137 (but not NaHS) also induced expression of PPARγ and ChREBP mRNA (Fig 4). Taken together, these results imply that exogenous H_2_S released from either GYY4137 or NaHS has a role to play in adipocyte differentiation.

### Inhibition of H_2_S production impairs adipogenesis-related gene activation.

**Fig 5:**
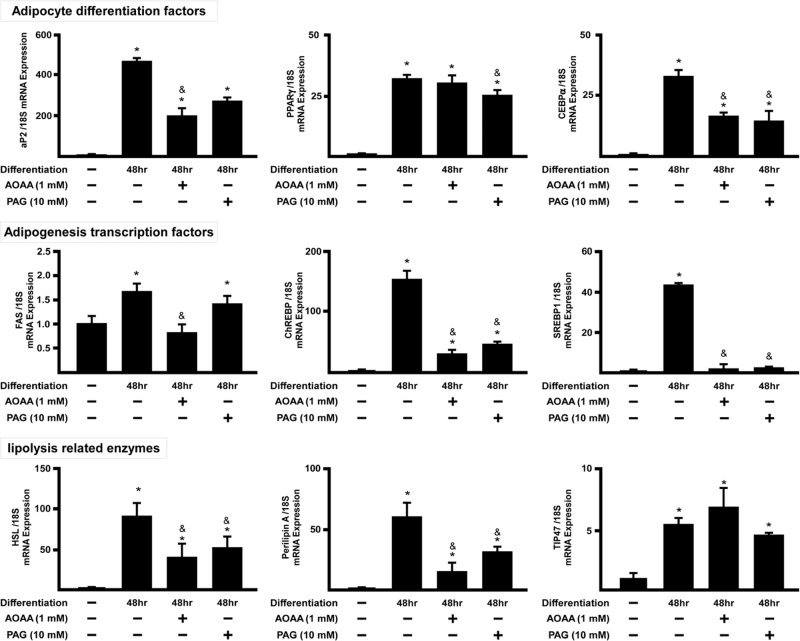
Inhibition of endogenous H_2_S synthesis with AOAA/PAG downregulates adipogenesis-related genes expression in 3T3L1 cells. 3T3L1 cells were co-incubated either with the AOAA (a CBS inhibitor) or PAG (a CSE inhibitor), for 2 days followed by real-time PCR assay of genes relevant to lipid metabolism. The relative mRNA expression levels were compared with the control group (normalized to 18S gene expression level) set as 1. Statistical significance was determined by ANOVA followed by Fisher's LSD posthoc analysis. Data shown are mean±SEM from 6 independent experiments. *P < 0.05 vs. negative control group; ^&^P < 0.05 vs. 48 h positive control group.

Since H_2_S donors promoted adipocyte differentiation we then determined whether endogenous H_2_S may also regulate adipogenesis and/or adipocyte maturation. To this end, we evaluated the effect of two pharmacological inhibitors of H_2_S-synthesizing enzymes i.e AOAA (CBS inhibitor) and PAG (CSE inhibitor) (Fig 5).

Expression of CEBPα, ChREBP, SREBP1, HSL and perilipin A mRNA expressions were significantly reduced by both AOAA and PAG. However, TIP47 mRNA expression was not altered by either AOAA or PAG suggesting that TIP47 gene is likely not a target for H_2_S (Fig 5).

### H_2_S regulates adipocyte differentiation by modulating FABP4/aP2 expression.

**Fig 6:**
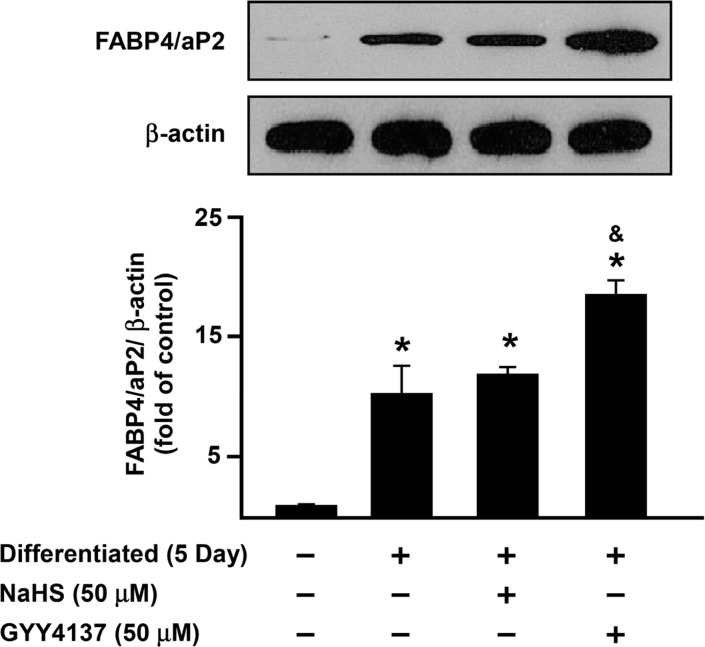
H_2_S upregulates FABP4/aP2 expression in 3T3L1 cells. 3T3L1 cells were incubated with either NaHS (50 μM) or GYY4137 (50 μM) for 5 days during differentiation, FABP4/aP2 expression was determined by Western Blotting. Data shows protein expression compared to the control group (normalized to β-actin) set as 1. Statistical significance was determined by ANOVA followed by Fisher's LSD posthoc analysis. Data shown are mean±SEM of 4 independent experiments. *P < 0.05 vs. negative control group; ^&^P < 0.05 vs. 5 days positive control group.

**Fig 7:**
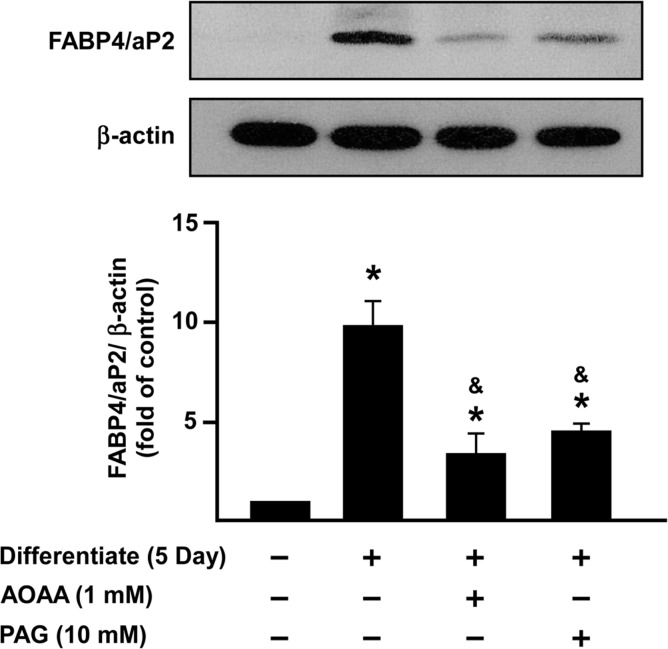
FABP4/aP2 protein expression is inhibited by AOAA and PAG treated 3T3L1 cells. 3T3L1 cells were incubated with either AOAA (1 mM) or PAG (10mM) for 5 days during differentiation, FABP4/aP2 expression was determined by Western Blotting. Data shows protein expression compared to the control group (normalized to β-actin) set as 1. Statistical significance was determined by ANOVA followed by Fisher's LSD posthoc analysis. Data shown are mean±SEM of 4 independent experiments. *P < 0.05 vs. negative control group; ^&^P < 0.05 vs. 5 days positive control group.

FABP4/aP2 is a key transcription factor in adipocyte differentiation [10,13,38] and a marker of adipogenesis [9,38]. To assess the effect of H_2_S on adipogenesis, 3T3L1 cells were incubated with or without GYY4137, NaHS, AOAA or PAG for 5 days and processed for Western blotting (Figs 6 and 7).

**Fig 8:**
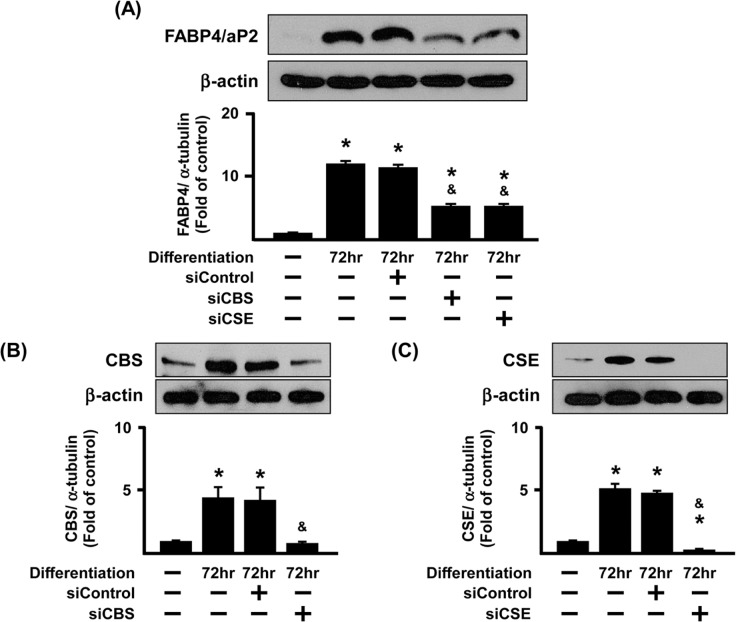
Effect of CBS and CSE knockdown on FABP4/aP2 protein expression in differentiated 3T3L1 cells. 3T3L1 cells were incubated with empty lipofectamine (siControl), siCBS (5 μg) or siCSE (5 μg) for 3 days during differentiation. Expression of FABP4/aP2, CBS and CSE protein expression was determined by Western Blotting. Data shows a representative blot (A) as well as protein expression compared to the control group and normalized to β-actin as 1 (B-D). Statistical significance was determined by ANOVA followed by Fisher's LSD posthoc analysis. Data shown are mean±SEM of 4 independent experiments. *P < 0.05 vs. negative control group; ^&^P < 0.05 vs. 3 days positive control group.

GYY4137, but not NaHS, increased FABP4/aP2 protein expression (Fig 6). Moreover, treatment of 3T3L1 cells with either AOAA or PAG suppressed FABP4/aP2 protein expression (Fig 7). Experiments were also conducted using siCSE or siCBS in adipocytes to knock down endogenous CSE or CBS and the expression of FABP4/aP2 was then determined 72 h after transfection (Fig 8).

In these experiments, siCBS or siCSE selectively reduced the expression of each enzyme respectively. Both treatments, like AOAA and PAG, suppressed FABP4/aP2 protein (Fig 8). Thus, endogenous H_2_S likely promotes adipogenesis by increasing FABP4/aP2 protein expression.

### H_2_S upregulates lipid accumulation by inhibiting lipolysis

**Fig 9:**
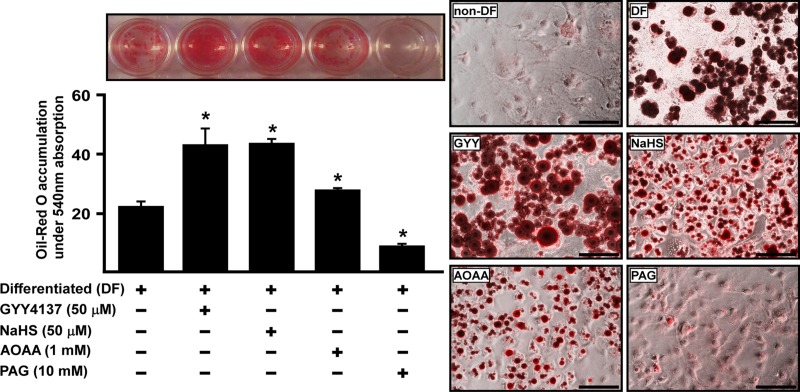
H_2_S promotes lipid accumulated in mature 3T3L1 adipocytes. 3T3L1 cells were incubated with either GYY4137 (50 μM), NaHS (50 μM), AOAA (1 mM) or PAG (10 mM) for 7 days of differentiation, lipid accumulation was determined by Oil-red O staining and well scanning (under 540 nm). Statistical significance was determined by ANOVA followed by Fisher's LSD posthoc analysis. Data shown are mean±SEM OF 6 independent experiments. *P < 0.05 vs. negative control group (non-DF); ^&^P < 0.05 vs. positive control group (DF).

**Fig 10:**
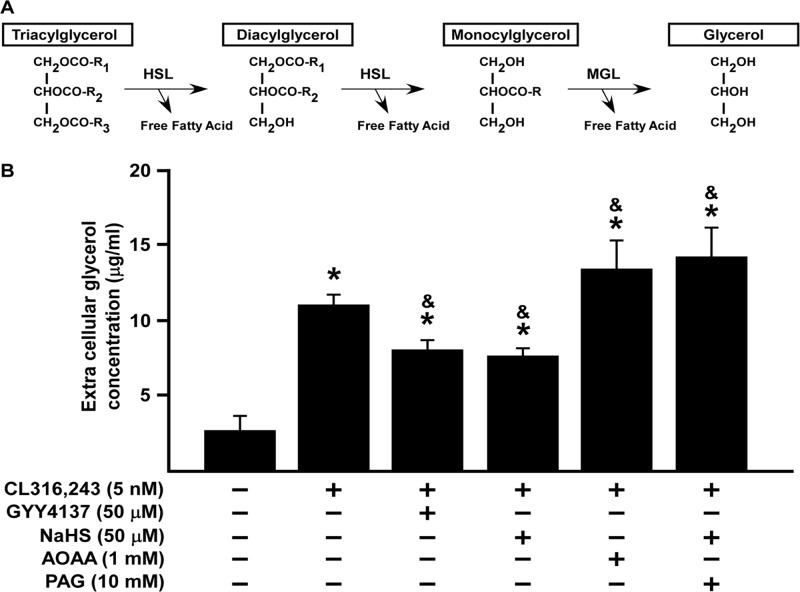
H_2_S inhibits lipolysis in matured 3T3L1 adipocyte. (A) The process of lipolysis. (B) Fully differentiated 3T3L1 cells were co-incubated with the β3-agonist, CL316,243 (5 nM) in the presence of absence of either GYY4137 (50 μM), NaHS (50 μM), AOAA (1 mM) or PAG (10 mM) for 1 h. Statistical significance was determined by ANOVA followed by Fisher's LSD posthoc analysis. Data shown are mean±SEM of 6 independent experiments. *P < 0.05 vs. negative control group; ^&^P < 0.05 vs. CL-316,243-treated.

To explore whether H_2_S affected the function of mature (c.f. differentiating) adipocytes, cells were first differentiated to adipocytes in the presence or absence of GYY4137, NaHS, AOAA or PAG for 7 days until the cells reached full maturation. Next, lipid droplets in mature adipocytes as well as glycerol content (an index of lipolysis), were examined by Oil Red O staining (Fig 9) and glycerol measurement (Fig 10) respectively.

As shown by Oil Red O staining, both GYY4137 and NaHS treatment significantly increased lipid accumulated in the cell bodies of mature adipocytes (Fig 9). In contrast, PAG reduced lipid accumulation in cells whilst, AOAA was without effect (Fig 9). These data suggest that change in endogenous H_2_S concentration contributes to modulation of adipocyte formation.

In order to probe further the effect of H_2_S in regulating adipocyte function, we assessed the effect of H_2_S donors and H_2_S synthesis inhibitors on glycerol release triggered by CL-361,234 (β-adrenoceptor agonist) incubation. During lipolysis, glycerol is released due to the breakdown of TG (Fig 10A). Exposure to CL-316,243 significantly increased glycerol concentration which effect was inhibited by treating cells with either GYY4137 or NaHS. In contrast, both AOAA and PAG augmented the ability of CL-316,243 to promote glycerol release. These results suggest that both exogenous and endogenous H_2_S modulates lipolysis in mature adipocytes.

## Discussion

Dysregulation of adipocyte proliferation, differentiation as well as disrupted adipocyte lipolysis contribute to obesity [5–8,14] but the role of H_2_S in this process is not clear. It has recently been shown that CSE is expressed in rat adipose tissues [21] and that H_2_S affects isoproterenol-stimulated lipolysis [24]. Moreover, CSE-knockout mice exhibit lower plasma cysteine and H_2_S concentrations as well as reduced body weight and white adipose tissue mass when compared with wild-type mice [39]. Each of these studies therefore point to a role for H_2_S in regulating adipocyte function. However, the detailed mechanism of action of H_2_S in adipogenesis and its effect on lipid homeostasis are not clear.

In the present study, we utilized 3T3L-1 cells, a well-established mouse preadipocyte cell-line, as a cellular model of adipogenesis [40]. Our results demonstrate that H_2_S is produced naturally during 3T3L1 differentiation. Moreover the expression of all three H_2_S -synthesisizing enzymes (i.e. CBS, CSE and 3-MST) was upregulated in a time-dependent manner during 3T3L1 cell differentiation. Thus, we propose firstly that H_2_S_,_ generated by one or more of these enzymes, plays a part in the process of adipocyte differentiation. To investigate the role of exogenous H_2_S in adipogenesis we evaluated the effect of the H_2_S donor agents, GYY4137 and NaHS, as well time-expired NaHS and ZYJ1122 as controls. The role of endogenous H_2_S was assessed in cells treated with either AOAA or PAG which inhibit CBS and CSE respectively by targeting the pyridoxal 5'-phosphate (PLP) binding sites [41,42] of each enzyme and by using siRNA for CSE and CBS. A number of gene markers of adipogenesis are known. These include transcription factors such as FABP4/aP2, PPARγ, CEBPα, FAS, ChREPB, SREBP1, HSL, perilipin A and TIP47 [8–12]. Among these, HSL, perilipin A and TIP47 are largely responsible for hydrolysis of TG into glycerol/FFA in mature adipocytes and as such are markers of adipocyte function [7,43]. Interestingly, GYY4137 (but not NaHS) promoted adipogenesis-related and lipolysis-related enzyme gene up-regulation (Fig 3). Moreover, compounds without H_2_S-releasing activity (time-expired NaHS and ZYJ1122) did not affect 3T3L1 differentiation-induced adipogenesis genes expression in 3T3L1 cells suggesting that H_2_S is indeed responsible for the effect of H_2_S donors on adipogenesis. GYY4137 was more effective than NaHS in these experiments presumably due to ability to release H_2_S slowly over a long period of time [37]. Intriguingly, both AOAA and PAG diminished adipogenesis-related genes expression during adipocytes differentiation. These results imply that both exogenous and naturally occurring H_2_S regulate 3T3L1 differentiation to adipocytes.

The present results also show that GYY4137 (but not NaHS) induced, whilst treatment with either AOAA or PAG, diminished FABP4/aP2 protein expression in differentiating 3T3L1 cells. That AOAA and PAG reduce FABP4/aP2 protein expression in these cells suggests a role for endogenous H_2_S in the differentiation process. However, AOAA and PAG inhibit CBS and CSE respectively by targeting the pyridoxal 5’ phosphate binding site on each of these enzymes and, as such, can at best be considered as non-selective inhibitors (reviewed in [24,44–46]). In this context, transfection of adipocytes with either siCBS or siCSE reduced expression of FABP4/aP2 thereby adding weight to the possibility that endogenous H_2_S regulates adipocyte function. That both exogenous and endogenous H_2_S regulates FABP4/aP2 expression is important since this protein is a fatty-acid transporter and binding protein critical for facilitating fatty acid uptake [10,38]. Indeed, deletion of FABP4/aP2 leads to embryonic lethality [47,48] whilst diminished FABP4/aP4 gene expression has been shown to protect animals from multiple metabolic syndromes including obesity, insulin resistance, hepatosteatosis and atherosclerosis [49–52]. That both endogenous and exogenous H_2_S promotes FABP4/aP2 expression supports a regulatory role for this gas in regulating adipocyte differentiation and raises the possibility that H_2_S donors may be of interest in the treatment of a range of metabolic diseases.

Lipid accumulation results illustrate that treatment with GYY4137 promoted adipogenesis by causing adipocyte hypertrophy, whilst NaHS upregulated lipid accumulation in mature adipocytes mainly by enhancing adipocyte maturation rate and stimulating adipocyte lipid droplet formation. These results suggest exogenously manipulating H_2_S levels directly enhance lipid accumulation in mature adipocytes. In contrast, diminished H_2_S production following treatment with either AOAA or PAG attenuates both the size and number of lipid droplets in mature adipocytes. Interestingly, PAG prevented lipid accumulation in lipid droplet suggesting that CSE plays a particularly important role in lipid droplets formation.

Mammalian cells store TG in lipid droplets to be hydrolyzed into fatty acids and glycerol when energetically desirable [6,7,13,35]. Any dysregulation of lipid synthesis and lipolysis contribute to accumulation of lipid droplets and promotes the development of obesity [5–8]. Moreover, the size of lipid droplets within the adipocyte is strongly correlated with the efficiency of adipolysis [5,6,35]. Since H_2_S plays a regulatory role in lipid droplet formation we next probed whether the H_2_S-regulated lipid accumulation is due to an effect on lipolysis rate. In the present study, we confirmed that treatment of mature adipocytes with either GYY4137 or NaHS significantly inhibited CL-316,243-induced adipolysis and that this was significantly enhanced by either AOAA or PAG. These data suggest that H_2_S directly regulates adipolysis in adipocytes.

Thus, we show here that that not only CSE but also CBS and 3-MST are highly expressed during adipogenesis. Moreover, these enzymes and H_2_S derived from their activity increase expression of adipogenesis-related genes and reduce adipolysis leading to accumulation of lipid droplets and triggering adipocyte hypertrophy. These data shed new light on the complex role of H_2_S in adipocyte biology and raise the possibility that drugs which manipulate endogenous H_2_S levels may have a role to play in metabolic disorders such as obesity.

## References

[1] Fantuzzi G, Mazzone T. Adipose tissue and atherosclerosis: exploring the connection. Arterioscler Thromb Vasc Biol. 2007; 27: 996–1003. 17303782 10.1161/ATVBAHA.106.131755

[2] Yanai H, Tomono Y, Ito K, Furutani N, Yoshida H, Tada N. The underlying mechanisms for development of hypertension in the metabolic syndrome. Nutr J. 2008; 7: 10 10.1186/1475-2891-7-10 18416854 PMC2335113

[3] Van Gaal LF, Mertens IL, De Block CE. Mechanisms linking obesity with cardiovascular disease. Nature. 2006;444: 875–880. 17167476 10.1038/nature05487

[4] Guilherme A, Virbasius JV, Puri V, Czech MP. Adipocyte dysfunctions linking obesity to insulin resistance and type 2 diabetes. Nat Rev Mol Cell Biol. 2008; 9: 367–377. 10.1038/nrm2391 18401346 PMC2886982

[5] Chiang JK, Koo M. Lipid accumulation product: a simple and accurate index for predicting metabolic syndrome in Taiwanese people aged 50 and over. BMC Cardiovasc Disord. 2012; 12: 78 10.1186/1471-2261-12-78 23006530 PMC3506496

[6] Langin D. Adipose tissue lipolysis as a metabolic pathway to define pharmacological strategies against obesity and the metabolic syndrome. Pharmacol Res. 2006; 53: 482–491. 16644234 10.1016/j.phrs.2006.03.009

[7] Lafontan M, Langin D. Lipolysis and lipid mobilization in human adipose tissue. Prog Lipid Res. 2009; 48: 275–297. 10.1016/j.plipres.2009.05.001 19464318

[8] Fajas L, Fruchart JC, Auwerx J. Transcriptional control of adipogenesis. Curr Opin Cell Biol. 1998; 10: 165–173. 9561840 10.1016/s0955-0674(98)80138-5

[9] Duplus E, Forest C. Is there a single mechanism for fatty acid regulation of gene transcription? Biochem Pharmacol. 2002; 64: 893–901. 12213584 10.1016/s0006-2952(02)01157-7

[10] Storch J, McDermott L. Structural and functional analysis of fatty acid-binding proteins. J Lipid Res. 2009; 50 Suppl: S126–131. 10.1194/jlr.R800084-JLR200 19017610 PMC2674722

[11] Barbier O, Torra IP, Duguay Y, Blanquart C, Fruchart JC, Glineur C, et al Pleiotropic actions of peroxisome proliferator-activated receptors in lipid metabolism and atherosclerosis. Arterioscler Thromb Vasc Biol. 2002; 22: 717–726. 12006382 10.1161/01.atv.0000015598.86369.04

[12] Shimano H. Sterol regulatory element-binding proteins (SREBPs): transcriptional regulators of lipid synthetic genes. Prog Lipid Res. 2001; 40: 439–452. 11591434 10.1016/s0163-7827(01)00010-8

[13] Shi Y, Burn P. Lipid metabolic enzymes: emerging drug targets for the treatment of obesity. Nat Rev Drug Discov. 2004; 3: 695–710. 15286736 10.1038/nrd1469

[14] Musri MM, Parrizas M. Epigenetic regulation of adipogenesis. Curr Opin Clin Nutr Metab Care. 2012; 15: 342–349. 10.1097/MCO.0b013e3283546fba 22617562

[15] Arner P. Human fat cell lipolysis: biochemistry, regulation and clinical role. Best Pract Res Clin Endocrinol Metab. 2005; 19: 471–482. 16311212 10.1016/j.beem.2005.07.004

[16] Li L, Rose P, Moore PK. Hydrogen sulfide and cell signaling. Annu Rev Pharmacol Toxicol. 2011; 51: 169–187. 10.1146/annurev-pharmtox-010510-100505 21210746

[17] Volpato GP, Searles R, Yu B, Scherrer-Crosbie M, Bloch KD, Ichinose F, et al Inhaled hydrogen sulfide: a rapidly reversible inhibitor of cardiac and metabolic function in the mouse. Anesthesiology. 2008; 108: 659–668. 10.1097/ALN.0b013e318167af0d 18362598 PMC2838418

[18] Skovgaard N, Gouliaev A, Aalling M, Simonsen U. The role of endogenous H_2_S in cardiovascular physiology. Curr Pharm Biotechnol. 2011; 12: 1385–1393. 22309020 10.2174/138920111798280956

[19] Wang R. Signaling pathways for the vascular effects of hydrogen sulfide. Curr Opin Nephrol Hypertens. 2011; 20: 107–112. 10.1097/MNH.0b013e3283430651 21301337

[20] Kolluru GK, Shen X, Kevil CG. A tale of two gases: NO and H_2_S, foes or friends for life? Redox Biol. 2013; 1: 313–318. 10.1016/j.redox.2013.05.001 24024166 PMC3757701

[21] Feng X, Chen Y, Zhao J, Tang C, Jiang Z, Geng B. Hydrogen sulfide from adipose tissue is a novel insulin resistance regulator. Biochem Biophys Res Commun. 2009; 380: 153–159. 10.1016/j.bbrc.2009.01.059 19166813

[22] Manna P, Jain SK. Hydrogen sulfide and L-cysteine increase phosphatidylinositol 3,4,5-trisphosphate (PIP3) and glucose utilization by inhibiting phosphatase and tensin homolog (PTEN) protein and activating phosphoinositide 3-kinase (PI3K)/serine/threonine protein kinase (AKT)/protein kinase Czeta/lambda (PKCzeta/lambda) in 3T3l1 adipocytes. J Biol Chem. 2011; 286: 39848–39859. 10.1074/jbc.M111.270884 21953448 PMC3220540

[23] Manna P, Jain SK. Vitamin D up-regulates glucose transporter 4 (GLUT4) translocation and glucose utilization mediated by cystathionine-gamma-lyase (CSE) activation and H_2_S formation in 3T3L1 adipocytes. J Biol Chem. 2012; 287: 42324–42332. 10.1074/jbc.M112.407833 23074218 PMC3516775

[24] Geng B, Cai B, Liao F, Zheng Y, Zeng Q, Fan X, et al Increase or decrease hydrogen sulfide exert opposite lipolysis, but reduce global insulin resistance in high fatty diet induced obese mice. PLoS One. 2013; 8: e73892 10.1371/journal.pone.0073892 24058499 PMC3772803

[25] Wang K, Peng H, Ni N, Dai C, Wang B. 2,6-dansyl azide as a fluorescent probe for hydrogen sulfide. J Fluoresc. 2014; 24: 1–5. 10.1007/s10895-013-1296-5 24081526

[26] Kanda T, Brown JD, Orasanu G, Vogel S, Gonzalez FJ, Sartoretto J, et al PPARgamma in the endothelium regulates metabolic responses to high-fat diet in mice. J Clin Invest. 2009; 119: 110–124. 10.1172/JCI36233 19065047 PMC2613459

[27] Tan EH, Hooi SC, Laban M, Wong E, Ponniah S, Wee A, et al CCAAT/enhancer binding protein alpha knock-in mice exhibit early liver glycogen storage and reduced susceptibility to hepatocellular carcinoma. Cancer Res. 2005; 65: 10330–10337. 16288022 10.1158/0008-5472.CAN-04-4486

[28] Reid BN, Ables GP, Otlivanchik OA, Schoiswohl G, Zechner R, Blaner WS, et al Hepatic overexpression of hormone-sensitive lipase and adipose triglyceride lipase promotes fatty acid oxidation, stimulates direct release of free fatty acids, and ameliorates steatosis. J Biol Chem. 2008; 283: 13087–13099. 10.1074/jbc.M800533200 18337240 PMC2442319

[29] Li J, Takaishi K, Cook W, McCorkle SK, Unger RH. Insig-1 "brakes" lipogenesis in adipocytes and inhibits differentiation of preadipocytes. Proc Natl Acad Sci USA. 2003; 100: 9476–9481. 12869692 10.1073/pnas.1133426100 PMC170943

[30] Le Lay S, Lefrere I, Trautwein C, Dugail I, Krief S. Insulin and sterol-regulatory element-binding protein-1c (SREBP-1C) regulation of gene expression in 3T3-L1 adipocytes. Identification of CCAAT/enhancer-binding protein beta as an SREBP-1C target. J Biol Chem. 2002; 277: 35625–35634. 12048207 10.1074/jbc.M203913200

[31] Kovsan J, Ben-Romano R, Souza SC, Greenberg AS, Rudich A. Regulation of adipocyte lipolysis by degradation of the perilipin protein: nelfinavir enhances lysosome-mediated perilipin proteolysis. J Biol Chem. 2007; 282: 21704–21711. 17488708 10.1074/jbc.M702223200

[32] Grasselli E, Voci A, Pesce C, Canesi L, Fugassa E, Gallo G, et al PAT protein mRNA expression in primary rat hepatocytes: Effects of exposure to fatty acids. Int J Mol Med. 2010; 25: 505–512. 20198297 10.3892/ijmm_00000370

[33] Zaheer S, Thangavel R, Wu Y, Khan MM, Kempuraj D, Zaheer A. Enhanced expression of glia maturation factor correlates with glial activation in the brain of triple transgenic Alzheimer's disease mice. Neurochem Res. 2013; 38: 218–225. 10.1007/s11064-012-0913-z 23086473 PMC3527678

[34] Iizuka K. Recent progress on the role of ChREBP in glucose and lipid metabolism. Endocr J. 2013; 60: 543–555. 23604004 10.1507/endocrj.ej13-0121

[35] Londos C, Sztalryd C, Tansey JT, Kimmel AR. Role of PAT proteins in lipid metabolism. Biochimie. 2005; 87: 45–49. 15733736 10.1016/j.biochi.2004.12.010

[36] Brasaemle DL. Thematic review series: adipocyte biology. The perilipin family of structural lipid droplet proteins: stabilization of lipid droplets and control of lipolysis. J Lipid Res. 2007; 48: 2547–2559. 17878492 10.1194/jlr.R700014-JLR200

[37] Lee ZW, Zhou J, Chen CS, Zhao Y, Tan CH, Li L, et al The slow-releasing hydrogen sulfide donor, GYY4137, exhibits novel anti-cancer effects in vitro and in vivo. PLoS One. 2011; 6: e21077 10.1371/journal.pone.0021077 21701688 PMC3119065

[38] Kralisch S, Fasshauer M. Adipocyte fatty acid binding protein: a novel adipokine involved in the pathogenesis of metabolic and vascular disease? Diabetologia. 2013; 56: 10–21. 10.1007/s00125-012-2737-4 23052058

[39] Mani S, Yang G, Wang R. A critical life-supporting role for cystathionine gamma-lyase in the absence of dietary cysteine supply. Free Radic Biol Med. 2011; 50: 1280–1287. 10.1016/j.freeradbiomed.2011.01.038 21310231

[40] Russell TR. Differentiation of 3T3-L2 fibroblasts into adipose cells in bromodeoxyuridine-suppressed cultures. Proc Natl Acad Sci USA. 1979; 76: 4451–4454. 92030 10.1073/pnas.76.9.4451 PMC411594

[41] Srilatha B, Adaikan PG, Li L, Moore PK. Hydrogen sulphide: a novel endogenous gasotransmitter facilitates erectile function. J Sex Med. 2007; 4: 1304–1311. 17655658 10.1111/j.1743-6109.2007.00561.x

[42] Whiteman M, Moore PK. Hydrogen sulfide and the vasculature: a novel vasculoprotective entity and regulator of nitric oxide bioavailability? J Cell Mol Med. 2009; 13: 488–507. 10.1111/j.1582-4934.2009.00645.x 19374684 PMC3822510

[43] Ducharme NA, Bickel PE. Lipid droplets in lipogenesis and lipolysis. Endocrinology. 2008; 149: 942–949. 10.1210/en.2007-1713 18202123

[44] Whiteman M, Winyard PG. Hydrogen sulfide and inflammation: the good, the bad, the ugly and the promising. Expert Rev Clin Pharmacol. 2011; 4: 13–32. 10.1586/ecp.10.134 22115346

[45] Fox B, Schantz JT, Haigh R, Wood ME, Moore PK, Viner N, et al Inducible hydrogen sulfide synthesis in chondrocytes and mesenchymal progenitor cells: is H_2_S a novel cytoprotective mediator in the inflamed joint? J Cell Mol Med. 2012; 16: 896–910. 10.1111/j.1582-4934.2011.01357.x 21679296 PMC3822858

[46] Asimakopoulou A, Panopoulos P, Chasapis CT, Coletta C, Zhou Z, Cirino G, et al Selectivity of commonly used pharmacological inhibitors for cystathionine beta synthase (CBS) and cystathionine gamma lyase (CSE). Br J Pharmacol. 2013; 169: 922–932. 10.1111/bph.12171 23488457 PMC3687671

[47] Schaffer JE, Lodish HF. Expression cloning and characterization of a novel adipocyte long chain fatty acid transport protein. Cell. 1994; 79: 427–436. 7954810 10.1016/0092-8674(94)90252-6

[48] Gimeno RE, Hirsch DJ, Punreddy S, Sun Y, Ortegon AM, Wu H, et al Targeted deletion of fatty acid transport protein-4 results in early embryonic lethality. J Biol Chem. 2003; 278: 49512–49516. 14512415 10.1074/jbc.M309759200

[49] Wymann MP, Schneiter R. Lipid signalling in disease. Nat Rev Mol Cell Biol. 2008; 9: 162–176. 10.1038/nrm2335 18216772

[50] Hotamisligil GS, Johnson RS, Distel RJ, Ellis R, Papaioannou VE, Spiegelman BM. Uncoupling of obesity from insulin resistance through a targeted mutation in aP2, the adipocyte fatty acid binding protein. Science. 1996; 274: 1377–1379. 8910278 10.1126/science.274.5291.1377

[51] Maeda K, Cao H, Kono K, Gorgun CZ, Furuhashi M, Uysal KT, et al Adipocyte/macrophage fatty acid binding proteins control integrated metabolic responses in obesity and diabetes. Cell Metab. 2005; 1: 107–119. 16054052 10.1016/j.cmet.2004.12.008

[52] Furuhashi M, Tuncman G, Gorgun CZ, Makowski L, Atsumi G, Vaillancourt E, et al Treatment of diabetes and atherosclerosis by inhibiting fatty-acid-binding protein aP2. Nature. 2007; 447: 959–965. 17554340 10.1038/nature05844 PMC4076119

